# Meaning in Life Is Associated with Differing Motivations to Use Social Networking Sites

**DOI:** 10.3390/bs16010120

**Published:** 2026-01-15

**Authors:** Roshan Rai, Mei-I Cheng, Jonathan Farnell

**Affiliations:** School of Applied Social Sciences, De Montfort University, Leicester LE1 9BH, UK; mcheng@dmu.ac.uk (M.-I.C.); jonathan.farnell@dmu.ac.uk (J.F.)

**Keywords:** Social Networking Sites, social media, meaning in life, wellbeing

## Abstract

Research often emphasises dysfunctional Social Networking Site (SNS) usage. In contrast, the current research examined a more positive element of human functioning, specifically how motivations to use SNSs may be associated with meaning in life, which can help give purpose and direction to people’s lives. A sample of 384 undergraduate students (aged 18 to 50; *M* = 20.95; *SD* = 4.95; 81.5% females) completed questionnaire-based measures of motivations to use SNSs, self-reported time spent on SNSs, and meaning in life (coherence, purpose, and mattering). Multiple regressions showed that models for coherence, purpose, and mattering explained 5.8–8.8% of the variance (*R*^2^ = 0.058–0.088). Self-expression was positively associated with coherence (β = 0.128), purpose (β = 0.16), and mattering (β = 0.137). Following/monitoring others predicted higher coherence (β = 0.158), and using SNSs to find information predicted higher purpose (β = 0.12). Academic purposes were positively related to mattering (β = 0.12). By contrast, using SNSs for new friendships predicted lower coherence (β = −0.197) and mattering (β = −0.154), entertainment predicted lower coherence (β = −0.178), and greater time on SNSs predicted lower purpose (β = −0.186). Overall, different motivations for using SNSs are associated with different facets of meaning in life.

## 1. Introduction

Social media and Social Networking Sites (SNSs) have become a ubiquitous feature of the modern world. An article for Forbes magazine (Wong, 2023) reported that there were an estimated 4.9 billion social media users on the planet in 2023, with the six most popular social media platforms being Facebook, YouTube, WhatsApp, Instagram, WeChat, and TikTok. However, the rise of social media has brought the question of whether such socially oriented technology enhances or damages our mental health (Gudka et al., 2021). Gudka et al. (2021) proposed a conceptual framework by which social media users could use social media to enhance their ability to thrive or *flourish* (with flourishing conceptualised as a psychological wellbeing construct), and the current research adopts a similar benefit-seeking approach to SNSs. The current research seeks to further understand the relationship between positive human functioning and SNSs by examining the relationships between judgements of meaning in life (which can help give direction and purpose to individuals) and how these might be associated with differing motivations to use SNSs (e.g., for social connectedness, social recognition, etc.) and self-reported time spent on SNSs.

### 1.1. Wellbeing, Meaning in Life, and Social Networking Sites

The question of how to conceptualise wellbeing is an age-old debate. Some argue that wellbeing is essentially hedonistic, consisting of feelings of pleasure and happiness, whilst others argue that wellbeing is eudaimonic, focusing on self-realisation and meaning (see Ryan & Deci, 2001). When it comes to meaning in life, some have seen this as somewhat different from, yet heavily related to, wellbeing (e.g., Steger, 2017). But to others, meaning in life has been firmly conceptualised as a wellbeing measure, albeit, a somewhat distinctive wellbeing measure (e.g., Costin & Vignoles, 2020). If accepted as a wellbeing measure, then meaning in life more easily fits into a eudaimonic framework. For instance, Huta and Waterman (2014) further argued that meaning was one of the four most-used concepts for researching eudaimonia (along with growth, authenticity, and excellence), and that meaning in life specifically was a near core marker of eudaimonic wellbeing.

Meaning in life has been described using a tripartite model consisting of coherence, purpose, and mattering (Costin & Vignoles, 2020). Coherence refers to the ability to make sense of one’s life and experiences. Purpose can be thought of as motivational, reflecting having aims in life or a guiding “vision”. Lastly, mattering refers to feelings of worth or having value that go beyond the “everyday” or “trivial” conditions of life. Costin and Vignoles (2020) developed four separate measures to assess meaning-in-life judgements: coherence, purpose, mattering, and a meaning-in-life subscale (which focuses exclusively on having meaning in life). They found that all four were somewhat distinct from each other, as well as somewhat distinct from related concepts (e.g., belonging, self-esteem, etc.).

As of yet, there is little research that directly measures meaning in life and SNS usage. Given that meaning in life helps people make sense of their lives and gives individuals guiding motivation (a life vision), it is very possible that meaning in life is also associated with using SNSs differently. In Gudka et al.’s (2021) systematic review of 118 social media studies, wellbeing was conceptualised as meaning/self-actualization (arguably measures that have some relation to meaning in life) in only four studies, with far more studies examining other wellbeing factors (e.g., relationships, subjective wellbeing, optimism/mastery, identity, etc.). Relatedly, a review by Meier and Reinecke (2023) further argued for the need to investigate the link between constructs such as meaning in life (as a dimension of eudaimonic wellbeing) and social media. Meier and Reinecke argued that the relationship between aspects of eudaimonic wellbeing, which includes meaning in life, and social media has largely been neglected in favour of hedonic wellbeing (e.g., life satisfaction, emotional affect) and measures of psychopathology and psychosocial risk/resilience (e.g., anxiety, self-esteem, etc.). The authors further argued that current research is largely lacking in direct measures of meaning in life, often measuring meaning in life indirectly. However, they do discuss evidence that much of the time spent on social media can be seen as meaningless, particularly when linked to escapism, procrastination, and hedonic satisfaction. Conversely, interacting with certain inspiring content (such as travel and nature imagery) was perceived as meaningful. Currently, there is a dearth of studies directly measuring meaning in life and social media. However, Li et al. (2023) did conduct one relevant study, finding that issues related to meaning in life (more specifically, meaning confusion, meaning anxiety, and meaning avoidance) in Chinese adolescents were associated with problematic social media usage (as well as problematic smartphone use and Internet gaming disorder). The question of how those with a clearer (more positive) meaning in life might use SNSs is still unanswered.

### 1.2. The Current Research

Whilst developing a scale to measure the different motivations to use SNSs, Pertegal et al. (2019) found relationships between these differing motivations and some wellbeing measures: loneliness was positively associated with the motivation to use SNSs for social connectedness and for self-expression, and life satisfaction was positively associated with using SNSs for finding information, academic purposes, social recognition, and following and monitoring others. Overall, it would seem that how and why people use social media matters with regard to the relationship between social media usage and measures of wellbeing. However, Pertegal et al. did not look at meaning-in-life measures, and there are reasons to suspect that specific motivations to use SNSs could be linked to specific aspects of meaning in life. For instance, perhaps motivations to use SNSs, such as to seek *information* or for *academic* reasons (as measured by Pertegal et al.’s (2019) scale) could be linked to the specific aspect of meaning in life *purpose* (as measured by Costin and Vignoles’ (2020) scale). It could well be argued that using SNSs for seeking information or for academic reasons inherently constitutes “purposeful” activity. In addition, SNSs also have the potential to provide an online social space for fostering meaningful social interactions and relationships. Overall, the varied capabilities of SNSs could potentially provide a flexible conduit for various purposeful and meaningful activities within cyberspace. Given these possibilities, the first purpose of this investigation is expressed as a research question:

Research Question 1 (RQ1): How are the different motivations to use SNSs related to the different measures of meaning in life?

Although RQ1 broadly investigates the different motivations to use SNSs and meaning in life, we can also make some specific predictions about certain motivations. Pertegal et al. (2019) did find that the motivation to use SNSs for social connectedness was positively associated with loneliness. However, numerous studies have linked social relationships to meaning in life. In a longitudinal study, Stavrova and Luhmann (2016) showed that meaning in life was both a source and a consequence of social connectedness. Given that this relationship exists offline, it would not be surprising if a similar relationship existed online and meaning in life was associated with using SNSs to connect with others. Furthermore, when considering the meaning in life subcomponents, there is reason to also think these might link to social connectedness: a sense of *purpose* has been linked to social support in older adults (Weston et al., 2021); social relationships have been shown to foster a sense of belonging (or *mattering* to others) that in turn is associated with greater overall reported meaning in life (Lambert et al., 2013); and using a photo elicitation methodology, school children most commonly cited friendships and *coherence* when thinking about their meaning in life (O’Rourke et al., 2019). Given this, it would not be surprising if meaning-in-life measures (meaning in life, purpose, mattering, and coherence) were associated with the motivation to use SNSs to connect with others.

**Hypothesis 1** **(H1):** *Motivations for using SNSs for social connection will be positively associated with meaning-in-life measures.*

There are also reasons to suspect that self-expression might be linked to meaning in life. As Waterman (1993) has argued, personal expressiveness is a function of the drive towards self-realisation (eudaimonic wellbeing) that can give meaning and direction to life. Furthermore, authentic living, which is in essence authentic self-expression, was found to be positively associated with the meaning in life subcomponents: purpose, comprehension (coherence), and mattering (Lutz et al., 2023). In addition, authentic self-presentation on social media has been found to be associated with positive mental health and increased wellbeing (see the review by Meier & Reinecke, 2023). We would therefore expect self-expression to be associated with meaning-in-life measures (meaning in life, purpose, mattering, and coherence), leading to the second hypothesis below.

**Hypothesis 2** **(H2):** *Motivations for using SNSs for self-expression will be positively associated with meaning-in-life measures.*

A further research question involves a measure of time spent on SNSs. Some research suggests that less time spent, but not necessarily no time, on social media is better for wellbeing (see Gudka et al., 2021). However, other research suggests that it is what people do on social media that is more important to wellbeing measures than the amount of time spent on social media (e.g., Rai et al., 2023). Furthermore, Meier and Reinecke’s (2023) review showed a mixed relationship between time spent on social media and meaning in life-related measures. So, again, the current research adopts a second research question to investigate the potential relationship between the amount of time spent on SNSs and meaning in life:

Research Question 2 (RQ2): How is time spent on SNSs related to the different measures of meaning in life?

## 2. Materials and Methods

### 2.1. Participants

Initially, a convenience sample of 400 undergraduate students completed an online survey. The data was collected between December of 2022 and March of 2023. In total, 16 participants were removed because they failed to finish the survey, leaving a final sample of 384 participants. All students were studying psychology at a university in the Midlands of England (UK) and participated for course credit. The sample comprised 313 females, 62 males, 8 non-binary/third gender participants, and 1 participant who did not report their gender. The participants’ ages ranged from 18 to 50 (mean = 20.95; *SD* = 4.95). Using Cohen’s conventions for multiple regression, an a priori G*Power analysis version 3.1 (Faul et al., 2009) with effect sizes in the small-to-medium range (f^2^ ≈ 0.08–0.15, α = 0.05, power = 0.80) and ten predictors indicated that medium effects (f^2^ = 0.15) require a sample of approximately 60–70 participants, while detecting effects closer to the small-to-medium range (f^2^ ≈ 0.08) requires a sample of around 110–120 participants. Our achieved sample of 384 participants, therefore, exceeds these conventional requirements by a substantial margin and provides high power to detect medium and small-to-medium effects, with reasonable sensitivity even to smaller effects.

### 2.2. Materials

*Demographics*: Participants were asked to report their age and gender.

*Hours on SNSs question*: Participants were asked the following single-item question: “Approximately how many hours per day do you spend using social networking sites?”, with responses made on the following scale: 0–1, 1–2, 2–3, 3–4, 4+. For data analysis purposes, 0–1 was scored as 1, 1–2 as 2, 2–3 as 3, 3–4 as 4, and 4+ as 5.

*Scale of Motives for Using Social Networking Sites (SMU-SNS)*: Participants’ different motives for using SNSs were measured by the 27-item SMU-SNS scale, which was developed by Pertegal et al. (2019). Using a 7-point Likert scale, participants were asked to report their motives for using SNSs. The scale measured nine motives for SNS usage, which all formed separate subscales (consisting of three items each) as follows: dating (α = 0.877), new friendships (α = 0.937), academic purposes (α = 0.85), social connectedness (α = 0.867), following and monitoring others (α = 0.756), entertainment (α = 0.919), social recognition (α = 0.815), self-expression (α = 0.872), and information (α = 0.886). Sample items include using SNSs “To stand out from others” (from the social recognition subscale) and “To keep up about what happens in the world” (from the information subscale).

*The Meaning in Life Scale*: Judgements of meaning in life were measured using a 16-item Likert scale developed by Costin and Vignoles (2020). Using a 7-point scale, participants were asked to report on aspects of their lives. There were four separate subscales (each subscale consisted of four items each): meaning in life (feeling that one’s life has meaning), coherence (being able to make sense of events in one’s life), purpose (having purpose and compelling life goals), and mattering (feeling that one mattered in the grand scheme of the Universe). Example items include “My life as a whole has meaning” (from the meaning in life subscale) and “I can make sense of the things that happen in my life” (from the coherence subscale). Each subscale had two items that were reverse scored. The following Cronbach’s alphas were obtained for the current sample: meaning in life judgements (α = 0.913), coherence (α = 0.816), purpose (α = 0.892), and mattering (α = 0.792).

### 2.3. Procedure

The current research was initially reviewed and approved by a university ethics committee. All testing took place anonymously online, using the research platform Qualtrics. All participants willing to participate signed consent forms. After completing the survey, participants were awarded course credit.

## 3. Results

Table 1 shows the means and standard deviations for all variables. Table 2 shows the zero-order correlations between variables.

Four regressions were conducted in order to determine whether the four different meaning-in-life subscales (meaning-in-life judgements, coherence, purpose, and mattering) could be predicted by the different motivations to use SNSs and self-reported hours spent on SNSs a day. Initially, tests of assumptions were carried out. Skewness and kurtosis values were examined to assess normality. Across all variables, skewness ranged from −1.24 to 1.74 and kurtosis ranged from −1.01 to 2.56. These values fall within commonly accepted thresholds for regression analysis, indicating no serious deviations from normality. There was no indication of multicollinearity, as all variation inflation factor (VIF) scores were less than 2 and ranged from 1.2 to 1.9. The assumption of independence of residuals was met as the Durbin–Watson values were approximately 2 for each regression model. There were no outliers, as all values for Cook’s distance were under 1.

### 3.1. Meaning in Life and SNSs

A multiple linear regression was conducted to determine whether meaning in life could be predicted from the different motivations to use SNSs and hours spent on SNSs per day. Using the enter method, a non-significant model was obtained: *F*(10, 373) = 1.23, *p* = 0.273.

### 3.2. Coherence and SNSs

A multiple linear regression was conducted to determine whether coherence could be predicted from the different motivations to use SNSs and hours spent on SNSs per day (see Table 3). Using the enter method, a significant model was obtained that explained 8.8% of the variance: *F*(10, 373) = 3.62, *p* < 0.001, with an *R^2^* of 0.088 (*R^2^* adjusted = 0.064). Self-expression (*B* = 0.096, β = 0.128, *p* = 0.032) and following and monitoring others (*B* = 0.125, β = 0.158, *p* = 0.016) positively predicted coherence, whilst new friendships (*B* = −0.136, β = −0.197, *p* = 0.001) and entertainment (*B* = −0.154, β = −0.178, *p* = 0.004) negatively predicted coherence. However, coherence was not predicted by the following: hours spent on SNSs per day (*B* = −0.08, β = −0.074, *p* = 0.172), dating (*B* = 0.046, β = 0.044, *p* = 0.419), academic purposes (*B* = −0.035, β = −0.046, *p* = 0.391), social connectedness (*B* = −0.039, β = −0.051, *p* = 0.453), social recognition (*B* = −0.043, β = −0.047, *p* = 0.415), and information (*B* = 0.084, β = 0.099, *p* = 0.097).

### 3.3. Purpose and SNSs

A multiple linear regression was conducted to determine whether purpose could be predicted from the different motivations to use SNSs and hours spent on SNSs per day (see Table 4). Using the enter method, a significant model was obtained that explained 8.1% of the variance: *F*(10, 373) = 3.3, *p* < 0.001, with an *R^2^* of 0.081 (*R^2^* adjusted = 0.057). Self-expression (*B* = 0.133, β = 0.16, *p* = 0.008) and information (*B* = 0.114, β = 0.12, *p* = 0.045) positively predicted purpose, whilst hours spent on SNSs per day negatively predicted purpose (*B* = −0.224, β = −0.186, *p* < 0.001). However, dating (*B* = 0.012, β = 0.01, *p* = 0.853), new friendships (*B* = −0.06, β = −0.077, *p* = 0.204), academic purposes (*B* = −0.029, β = −0.035, *p* = 0.516), social connectedness (*B* = 0.005, β = 0.006, *p* = 0.927), following and monitoring others (*B* = −0.018, β = −0.02, *p* = 0.759), entertainment (*B* = −0.040, β = −0.041, *p* = 0.506), and social recognition (*B* = −0.097, β = −0.095, *p* = 0.103) did not predict purpose.

### 3.4. Mattering and SNSs

A multiple linear regression was conducted to determine whether mattering could be predicted from the different motivations to use SNSs and hours spent on SNSs per day (see Table 5). Using the enter method, a significant model was obtained that explained 5.8% of the variance: *F*(10, 373) = 2.29, *p* = 0.013, with an *R^2^* of 0.058 (*R^2^* adjusted = 0.032). Self-expression (*B* = 0.116, β = 0.137, *p* = 0.024) and academic purposes (*B* = 0.102, β = 0.121, *p* = 0.029) positively predicted mattering, whilst new friendships negatively predicted mattering (*B* = −0.12, β = −0.154, *p* = 0.013). However, mattering was not predicted by the following: hours spent on SNSs per day (*B* = −0.057, β = −0.047, *p* = 0.398), dating (*B* = −0.057, β = −0.048, *p* = 0.388), social connectedness (*B* = −0.034, β = −0.04, *p* = 0.563), following and monitoring others (*B* = −0.015, β = −0.017, *p* = 0.798), entertainment (*B* = −0.095, β = −0.096, *p* = 0.127), social recognition (*B* = 0.018, β = 0.017, *p* = 0.773), and information (*B* = 0.012, β = 0.12, *p* = 0.84).

## 4. Discussion

In answer to the first research question (RQ1), motivations to use SNSs were found to be related to a number of meaning-in-life measures. The motivations to use SNSs for self-expression and following and monitoring others were positively associated with coherence, whilst the motivations to use SNSs for new friendships and entertainment were negatively associated with coherence. The motivations for using SNSs for self-expression and finding information were positively associated with purpose. The motivations for using SNSs for self-expression and academic purposes were positively associated with mattering, whilst using SNSs for new friendships was negatively related to mattering. However, perhaps surprisingly, the separate subscale measuring meaning-in-life judgements (independent of coherence, purpose, and mattering) was unrelated to any motivation to use SNSs. As the motivation to use SNSs for social connection was unrelated to any meaning-in-life measure, H1 was rejected. However, there was partial support for H2, as using SNSs for self-expression was positively associated with coherence, purpose, and mattering but not the separate meaning-in-life subscale. In answer to the second research question (RQ2), self-reported hours spent on SNSs a day were significantly and negatively associated with purpose only. No other meaning-in-life measure was related to self-reported hours spent on SNSs.

Previous research by Li et al. (2023) found that issues with meaning in life in Chinese adolescents were associated with problematic social media usage. In contrast, the current research shows that social networking is not always associated with negative outcomes and can also be associated with positive outcomes. In keeping with Gudka et al.’s (2021) theoretical framework, the current study adds support to the notion that SNSs can be associated with human flourishing. In accordance with Meier and Reinecke (2023), the current research helps to address the need for more research by directly looking at social media and meaning in life (eudaimonic wellbeing). Although the amount of variance explained in the current research was relatively modest, we would argue that the pattern of results is theoretically meaningful and suggests that specific motives for SNS use are linked to different facets of meaning in life.

Interestingly, being motivated to use SNSs for self-expression was positively associated with three measures—coherence, purpose, and mattering. No other SNS measure was as frequently associated with positive measures. The question of why this particular motivation was so often linked to meaning-in-life measures is quite intriguing. From previous research, self-expressiveness has been shown to be important to eudaimonic aspects of wellbeing (Waterman, 1993; Waterman et al., 2008). Additionally, authentic living (essentially a facet of authentic self-expression) was positively associated with the meaning in life subcomponents: purpose, comprehension (coherence), and mattering (Lutz et al., 2023). So, a link between self-expression/authenticity has been noted in the literature. Furthermore, like previous research, the current study also shows a link between the motivation for self-expression (as specifically focused on SNSs) and multiple measures of wellbeing (meaning-in-life measures). However, and perhaps curiously, Pertegal et al.’s (2019) original paper validating the SMU-SNS found that loneliness was positively related to self-expression. It is possible that both negative factors (loneliness) and positive factors (coherence, purpose, and mattering) could lead to, or derive from (as causality cannot be inferred from the current methodology), self-expression on SNSs. Or, it could be the case that the specific manner in which people self-express on SNSs makes a difference. Meier and Reinecke’s (2023) review found that authentic self-presentation was linked to higher wellbeing, although the current research did not employ a methodology sensitive enough to measure the authenticity of self-expression. Further research is needed to tease out the exact relationships and mechanisms behind these associations.

In addition to self-expression, other motivations to use SNSs were positively related to meaning-in-life measures. Following and monitoring others was positively associated with coherence, information was positively associated with purpose, and academic purposes were positively associated with mattering. This all adds to a profile of SNS users who have specific motivations to use SNSs, and who also report higher wellbeing scores (in this case, meaning-in-life measures). These findings support Pertegal et al.’s (2019) research, in that they also found that using SNSs for information, academic purposes, and following and monitoring others was associated with the wellbeing measure satisfaction with life. But also, more broadly, these findings support those (e.g., Gudka et al., 2021; Rai et al., 2023) who argue that social media usage can be associated with positive outcomes, depending on how the user interacts with SNSs.

Although some motivations to use SNSs were associated with higher wellbeing, it was also the case that some variables were negative predictors of wellbeing measures. These negative relationships could well be a reflection of unmet needs, or even underlying distress. As Martela and Steger (2016) have argued, meaning in life (as measured by the tripart model of meaning in life) is important to positive human functioning, with a lack of meaning in life potentially being associated with uncertainty/incomprehensibility (the opposite of coherence), a loss of direction/aimlessness (the opposite of purpose), and a lack of worth (the opposite of significance/mattering). In the current study, the motivation to use SNSs for entertainment was negatively associated with coherence. This might not be so surprising given that social media usage has been found to be related to both boredom and lower subjective wellbeing measures (Bai et al., 2021). A second motivation to use SNSs that was negatively associated with wellbeing was the motivation for new friendships, which was negatively predictive of both coherence and purpose. This finding might mirror Pertegal et al.’s (2019) previous finding that loneliness was positively related to the motivation to use SNSs for social connectedness. Intriguingly, it has been shown that using social media to socially interact with others can be associated with higher wellbeing (Rai et al., 2023) and lower loneliness (Yang, 2016). There is an obvious question of how to resolve these seemingly contrary findings. A motivation measured in the current study, like making new friendships through SNSs, does not necessarily reflect behaviour. The scales used in Rai et al. (2023) and Yang’s (2016) research asked participants to report how much they used SM to interact with others, whilst, Pertegal et al.’s (2019) SMU-SNS asked participants to report their motivations, not behaviours. A motivation for social contact does not necessarily mean that a user has enough social contact, which could possibly help account for these discrepant findings, but further research is needed to investigate this possibility.

Hours spent on SNSs a day were another factor that was negatively associated with wellbeing measures—specifically, purpose. Given the association-based nature of the current research, the exact mechanisms behind the relationship between hours spent on SNSs and purpose cannot be determined with certainty, though Meier and Reinecke’s (2023) review found that screen time could be linked to a sense of meaninglessness and a lack of purpose. Additionally, previous findings have found that more time spent on social media can be linked with negative outcomes (see Gudka et al., 2021).

One perhaps surprising finding, which is in contrast to H1, was that social connection was not associated with any measure of meaning in life. Pertegal et al. (2019) did find that loneliness was positively associated with the motivation to use SNSs for social connection, which might throw some doubt on H1. Although, given that much previous research has found that social connection has been linked to meaning in life (e.g., Lambert et al., 2013; Lutz et al., 2023; Stavrova & Luhmann, 2016), we had still anticipated a relationship between using SNSs for social connection and meaning in life. Even though this association was absent, it should be noted that other social relationship measures (aside from using SNSs for social connection) were associated with meaning in life. Firstly, the motivation to use SNSs for following and monitoring others was positively associated with coherence, and secondly, using SNSs to make new friendships was negatively linked to mattering.

### 4.1. Further Implications

The current research challenges the notion that SNSs are destined to be associated with dysfunction and harm. Although SNSs can undoubtedly be linked with negative outcomes, the current research provides evidence that this is not always the case. Instead, the motivations to use SNSs can be more important to wellbeing than time spent on SNSs. Given the current research, users could be more mindful of why they want to use SNSs. Using SNSs for self-expression, information seeking, and academic purposes could bring psychological rewards, whilst using SNSs for entertainment might not be as conducive to wellbeing. These insights could also be of benefit to those working with SNSs, such as those designing SNS platforms. Additionally, educational institutions could also be mindful that people are motivated to use SNSs for academic purposes and finding information. Lastly, for mental health practitioners working with those struggling with their SNS usage, it could be valuable to consider not just the time their clients spend on SNSs, but also their clients’ motivations to use SNSs, as the current research demonstrates that some motivations to use SNSs are linked to higher reported wellbeing.

### 4.2. Limitations and Future Research

The association-based methodology utilised in the current research has the typical limitations of this type of methodology: causality is difficult to ascertain, and the exact mechanisms behind the relationships identified are hard to determine. It would be useful for future research to use methodologies that could better identify causal mechanisms. For example, research could employ methodologies asking participants to restrict their SNS usage (in a similar manner to research by Hartanto et al., 2025). Or, research could attempt to modify SNS users’ perceptions and behaviours (as has been performed in intervention studies, such as in Hou et al., 2019), perhaps asking them to focus on more meaningful SNS interaction (e.g., focusing on self-expression, etc.). Any changes in participants’ meaning-in-life scores after making changes to their SNS usage could then be noted (and causality more confidently derived). Furthermore, it would be interesting for future research to investigate other possibly related wellbeing/meaning-in-life factors with SNS usage. For instance, previous research has shown that self-expressiveness was related to self-realisation motivations (Waterman, 1993; Waterman et al., 2008), and it could be further worthwhile to investigate self-expression along with self-realisation motivations in a social media context.

The current research also recruited psychology undergraduate samples from a Western/UK university. Although in line with guidelines from our ethics board (to minimise demographic data collection unless it is directly related to the research questions), we did not collect further demographic information aside from gender and age (e.g., such as nationality, ethnicity, or first language). This presents a limitation of the current research. Furthermore, the sample was heavily weighted towards females (313 of the 384 participants), which is most likely reflective of the tendency for more females to study psychology than males. Overall, there needs to be caution in regard to the generalisability of the current sample, and future research could investigate SNS users and meaning in life in more diverse populations (e.g., non-student, non-Western, etc.), more accurately measure demographic data, and recruit greater proportions of male participants.

Further limitations include the somewhat crude measure of time spent on SNSs used in the current research. Although much of the previous research has utilised self-reported time spent on SNSs/social media, it has been pointed out that self-reported time spent on SNSs/social media can be somewhat inaccurate, sometimes differing from what might be recorded by an electronic device (Kaye, 2022). Additionally, it could be argued that the measure of motivations to use SNSs used in the current research also lacks specificity, as the questions were not directed to a particular SNS platform (e.g., like Facebook, Instagram, etc.). As SNSs can vary substantially in their forms (e.g., focus on pictures as opposed to written text, etc.), any interplay between the type of SNSs and motivations to use them cannot be examined in the current research.

Future work could also include covariates to further test the robustness of the relationships in the current study. For example, it has been found that those at later life stages reported more presence of meaning in life, whilst those in earlier life stages reported greater search for meaning (Steger et al., 2009). Although any analysis of age in the current research would be greatly hampered, as our sample comprised university students mostly in the early stages of life, future research could examine the interplay of the lifespan with meaning in life and motivations to use SNSs. Furthermore, other covariates could be investigated. For example, other wellbeing variables (such as happiness and positive affect) have been shown to be associated with meaning in life (e.g., Steger et al., 2009). Future research could investigate any interplay between meaning-in-life scores, other wellbeing scores, and motivations to use SNSs.

## 5. Conclusions

The current research took a relatively novel approach by looking at the relationship between SNSs and meaning in life. Overall, certain motivations to use SNSs (e.g., for self-expression) were found to be associated with higher self-reported levels of certain meaning-in-life measures (e.g., purpose), whilst other motivations to use SNSs (e.g., using SNSs for new friendships) and time spent on SNSs were found to be negatively associated with certain meaning-in-life measures (e.g., mattering). This presents a more nuanced picture of SNS usage and wellbeing, in that those scoring higher in meaning in life are motivated to use SNSs in particular ways, just as those lower in meaning in life are motivated to use SNSs in different ways.

## Figures and Tables

**Table 1:** Mean and standard deviations for all variables.

	Means	*SD*
Hours on SNSs per day	3.87	1.13
Coherence	4.65	1.22
Purpose	5.03	1.36
Mattering	4.34	1.38
Meaning in life	5.22	1.36
Academic purposes	3.96	1.63
Dating	1.74	1.17
Entertainment	5.71	1.4
Following and monitoring others	4.34	1.54
Information	5.33	1.43
New friendships	3.25	1.76
Self-expression	3.41	1.63
Social connectedness	4.75	1.6
Social recognition	2.31	1.34

**Table 2:** Zero-order correlations for all variables.

	1	2	3	4	5	6	7	8	9	10	11	12	13
1. Meaning in life	-												
2. Coherence	0.562 **	-											
3. Purpose	0.586 **	0.534 **	-										
4. Mattering	0.715 **	0.465 **	0.476 **	-									
5. Hours on SNSs per day	−0.076	−0.107 *	−0.19 **	−0.078	-								
6. Dating	−0.093	−0.019	−0.055	−0.091	0.18 **	-							
7. New friendships	−0.073	−0.16 **	−0.085	−0.131 *	0.202 **	0.381 **	-						
8. Academic purposes	−0.004	−0.081	−0.061	0.063	0.162 **	0.059	0.241 **	-					
9. Social connectedness	−0.002	−0.068	−0.022	−0.075	0.2 **	0.148 **	0.409 **	0.32 **	-				
10. Following and monitoring others	−0.01	0.029	−0.055	−0.049	0.253 **	0.117 *	0.246 **	0.309 **	0.567 **	-			
11. Entertainment	0.001	−0.128 *	−0.063	−0.081	0.311 **	−0.073	0.072	0.264 **	0.42 **	0.454 **	-		
12. Social recognition	−0.032	−0.045	−0.088	0.006	0.164 **	0.185 **	0.314 **	0.225 **	0.26 **	0.353 **	0.159 **	-	
13. Self-expression	0.063	0.069	0.088	0.076	0.191 **	0.111 *	0.304 **	0.195 **	0.337 **	0.312 **	0.167 **	0.416 **	-
14. Information	0.095	0.045	0.097	−0.002	0.149 **	−0.012	0.177 **	0.231 **	0.418 **	0.336 **	0.393 **	0.117 **	0.368 **

* *p* < 0.05; ** *p* < 0.01.

**Table 3:** Summary of multiple regression analysis for variables predicting coherence.

	*B*	SE	β
Constant	5.3	0.331	
Dating	0.046	0.057	0.044
New friendships	−0.136	0.042	−0.197 **
Academic	−0.035	0.04	−0.046
Social connectedness	−0.039	0.052	−0.051
Following and monitoring others	0.125	0.052	0.158 *
Entertainment	−0.154	0.054	−0.178 **
Social Recognition	−0.043	0.053	−0.047
Self-expression	0.096	0.045	0.128 *
Information	0.084	0.051	0.099
Hours spent on SNSs per day	−0.08	0.058	−0.074

* *p* < 0.05, ** *p* < 0.01.

**Table 4:** Summary of multiple regression analysis for variables predicting purpose.

	*B*	SE	β
Constant	5.62	0.371	
Dating	0.012	0.064	0.01
New friendships	−0.06	0.047	−0.077
Academic	−0.029	0.045	−0.035
Social connectedness	0.005	0.058	0.006
Following and monitoring others	−0.018	0.058	−0.02
Entertainment	−0.04	0.06	−0.041
Social recognition	−0.097	0.059	−0.095
Self-expression	0.133	0.05	0.16 **
Information	0.114	0.057	0.12 *
Hours spent on SNSs per day	−0.224	0.065	−0.186 ***

* *p* < 0.05, ** *p* < 0.01, *** *p* < 0.001.

**Table 5:** Summary of multiple regression analysis for variables predicting mattering.

	*B*	SE	β
Constant	4.92	0.381	
Dating	−0.057	0.066	−0.048
New friendships	−0.12	0.048	−0.154 *
Academic	0.102	0.047	0.121 *
Social connectedness	−0.034	0.06	−0.04
Following and monitoring others	−0.015	0.06	−0.017
Entertainment	−0.095	0.062	−0.096
Social recognition	0.018	0.061	0.017
Self-expression	0.116	0.051	0.137 *
Information	0.012	0.058	0.012
Hours spent on SNSs per day	−0.057	0.067	−0.047

* *p* < 0.05.

## Data Availability

The data that supports the findings of this study is available at https://doi.org/10.21253/DMU.24026394.
